# Evolution of Lysine Biosynthesis in the Phylum *Deinococcus-Thermus*

**DOI:** 10.1155/2012/745931

**Published:** 2012-05-08

**Authors:** Hiromi Nishida, Makoto Nishiyama

**Affiliations:** ^1^Agricultural Bioinformatics Research Unit, Graduate School of Agricultural and Life Sciences, University of Tokyo, Bunkyo-ku, Tokyo 113-8657, Japan; ^2^Biotechnology Research Center, University of Tokyo, Bunkyo-ku, Tokyo 113-8657, Japan

## Abstract

*Thermus thermophilus* biosynthesizes lysine through the **α**-aminoadipate (AAA) pathway: this observation was the first discovery of lysine biosynthesis through the AAA pathway in archaea and bacteria. Genes homologous to the *T. thermophilus* lysine biosynthetic genes are widely distributed in bacteria of the *Deinococcus-Thermus* phylum. Our phylogenetic analyses strongly suggest that a common ancestor of the *Deinococcus-Thermus* phylum had the ancestral genes for bacterial lysine biosynthesis through the AAA pathway. In addition, our findings suggest that the ancestor lacked genes for lysine biosynthesis through the diaminopimelate (DAP) pathway. Interestingly, *Deinococcus proteolyticus* does not have the genes for lysine biosynthesis through the AAA pathway but does have the genes for lysine biosynthesis through the DAP pathway. Phylogenetic analyses of *D. proteolyticus* lysine biosynthetic genes showed that the key gene cluster for the DAP pathway was transferred horizontally from a phylogenetically distant organism.

## 1. Introduction

The *Deinococcus-Thermus* phylum constitutes one of the major bacterial evolutionary lineages [1, 2]. At present, the genome sequence data of 6 genera (13 organisms) belonging to this phylum are available in the Kyoto Encyclopedia of Genes and Genomes (KEGG) database [3].

Two pathways for lysine biosynthesis have been described, namely, the *α*-aminoadipate (AAA) pathway and the diaminopimelate (DAP) pathway [5]. The AAA pathway has two different types [6]. In *T. thermophilus*, a gene cluster was found for lysine biosynthesis not through the DAP pathway but through the AAA pathway [6–8]. Although *Deinococcus radiodurans* has genes homologous to the *T. thermophilus* lysine biosynthetic genes, these genes are scattered on the genome [9]. In addition, the *D. radiodurans* aspartate kinase that catalyzes the phosphorylation of l-aspartate (the first reaction in the DAP pathway) is structurally and phylogenetically very different from that of *T. thermophilus* [10]. Recent studies have shown that the genome signatures of these 2 bacteria are different [4], supporting the theory that *Deinococcus* species acquired genes from various other bacteria to survive different kinds of environmental stresses, whereas *Thermus* species have acquired genes from thermophilic bacteria to adapt to high-temperature environments [11].

 The distribution of lysine biosynthetic genes in the *Deinococcus-Thermus* phylum has not been clearly described. In this study, we compared the distribution of the genes for lysine biosynthesis between 13 organisms (*D. deserti*, *D. geothermalis*, *D. maricopensis*, *D. proteolyticus*, *D. radiodurans*, *Marinithermus hydrothermalis*, *Meiothermus ruber*, *M. silvanus*, *Oceanithermus profundus*, *T. scotoductus*, *T. thermophilus* HB8, *T. thermophilus* HB27, and *Truepera radiovictrix*).

## 2. Methods

We analyzed the distribution of each of the following 10 enzymes related to lysine biosynthesis through the AAA pathway in the *Deinococcus-Thermus* phylum: *α*-aminoadipate aminotransferase, homoisocitrate dehydrogenase, LysW-*γ*-l-lysine aminotransferase, LysW-*γ*-l-lysine hydrolase, LysW-*γ*-l-*α*-aminoadipate kinase, LysW-*γ*-l-*α*-aminoadipyl-6-phosphate reductase, *α*-aminoadipate-LysW ligase LysX, LysU, LysT, and homocitrate synthase. In addition, we analyzed the distribution of each of the following 6 enzymes related to lysine biosynthesis through the DAP pathway: aspartate kinase, aspartate-semialdehyde dehydrogenase, dihydrodipicolinate synthase, dihydrodipicolinate reductase, ll-diaminopimelate aminotransferase, and diaminopimelate decarboxylase.

 Homologous genes were selected on the basis of BLASTp search results by using each *T. thermophilus* enzyme for lysine biosynthesis through the AAA pathway and each *D. proteolyticus* enzyme for lysine biosynthesis through the DAP pathway. Multiple alignments were obtained using 20 amino acid sequences, with the highest to the 20th highest score by the BLASTp result. Maximum-likelihood trees were constructed using MEGA software version 5 [12]. The WAG model [13] was used as the amino acid substitution model. The nearest neighbor interchange was used for the maximum-likelihood heuristic method. The *γ*-distributed rate was considered, and the number of discrete *γ* categories was 3. Bootstrap analysis was performed with 100 replicates. 

## 3. Results and Discussion

Genes homologous to the *T. thermophilus* genes for lysine biosynthesis through the AAA pathway were found to be widely distributed in bacteria belonging to the *Deinococcus-Thermus* phylum, except for *D. proteolyticus* (Table 1). Among the 13 organisms examined, *Marinithermus*, *Oceanithermus*, and *Truepera* have the largest gene cluster, containing 8 lysine biosynthetic genes (Table 1). In each phylogenetic analysis of the 10 enzymes, lysine biosynthetic genes of the *Deinococcus-Thermus* phylum were found to have a common ancestor (See in Supplementary Material Figures S1−S10 available online at doi:10.1155/2012/745931). We hypothesize that a common ancestor of the *Deinococcus-Thermus* phylum biosynthesized lysine through the AAA pathway.

 In contrast, the distribution of genes for lysine biosynthesis through the DAP pathway was found to be limited in the *Deinococcus-Thermus* phylum (Table 2). Thus, ll-diaminopimelate aminotransferase and dihydrodipicolinate reductase were identified in no bacteria other than *D. proteolyticus* (Table 2). This observation supports our hypothesis that a common ancestor of the *Deinococcus-Thermus* phylum biosynthesized lysine not through the DAP pathway, but through the AAA pathway.

**Figure 1:**
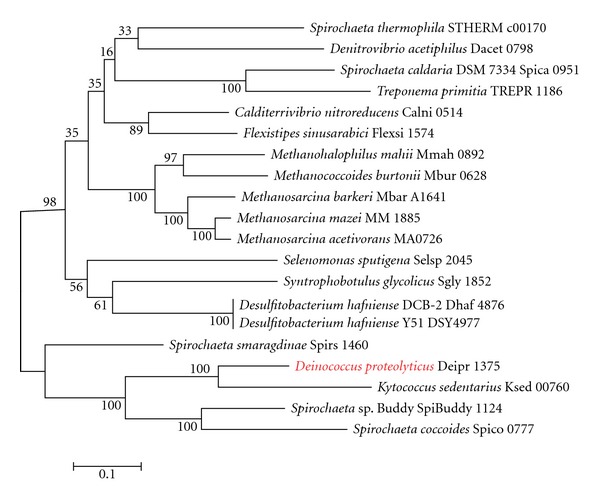
Phylogenetic relationship between *Deinococcus proteolyticus* diaminopimelate decarboxylase and related proteins. Multiple alignment was obtained using the top 20 amino acid sequences of the BLASTp search result for *D. proteolyticus* diaminopimelate decarboxylase (Deipro 1375), as based on the Kyoto Encyclopedia of Genes and Genomes (KEGG) database. The maximum-likelihood tree was constructed using MEGA software version 5 [12]. The WAG model was used as the amino acid substitution model. The nearest neighbor interchange was used for the maximum-likelihood heuristic method. The *γ*-distributed rate was considered, and the number of discrete *γ* categories was 3. Bootstrap analysis was performed with 100 replicates. Red indicates *D. proteolyticus*.

**Figure 2:**
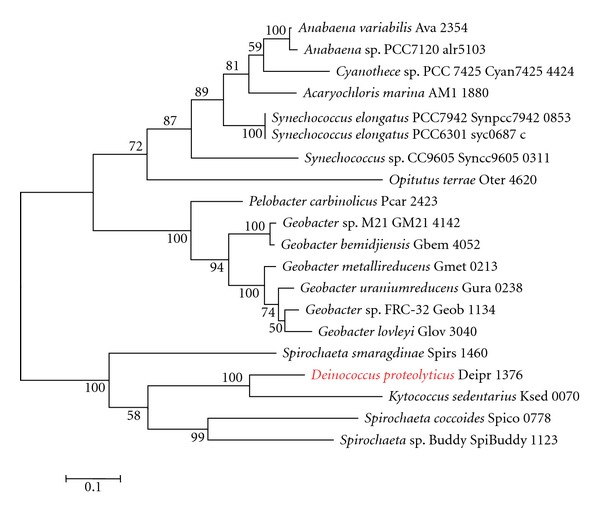
Phylogenetic relationship between *Deinococcus proteolyticus* ll-diaminopimelate aminotransferase and related proteins. Multiple alignment was obtained using the top 20 amino acid sequences of the BLASTp search result for *D. proteolyticus* ll-diaminopimelate aminotransferase (Deipro 1376), as based on the KEGG database. The maximum-likelihood tree was constructed using MEGA software version 5 [12]. The WAG model was used as the amino acid substitution model. The nearest neighbor interchange was used for the maximum-likelihood heuristic method. The *γ*-distributed rate was considered, and the number of discrete *γ* categories was 3. Bootstrap analysis was performed with 100 replicates. Red indicates *D. proteolyticus*.

**Figure 3:**
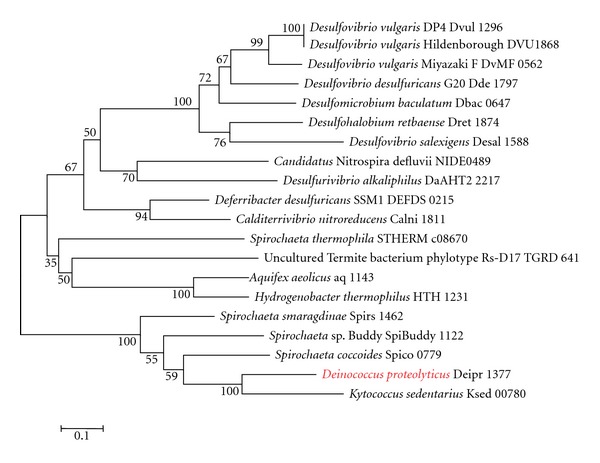
Phylogenetic relationship between *Deinococcus proteolyticus* dihydrodipicolinate synthase and related proteins. Multiple alignment was obtained using the top 20 amino acid sequences of the BLASTp search result for *D. proteolyticus* dihydrodipicolinate synthase (Deipro 1377), as based on the KEGG database. The maximum-likelihood tree was constructed using MEGA software version 5 [12]. The WAG model was used as the amino acid substitution model. The nearest neighbor interchange was used for the maximum-likelihood heuristic method. The *γ*-distributed rate was considered, and the number of discrete *γ* categories was 3. Bootstrap analysis was performed with 100 replicates. Red indicates *D. proteolyticus*.

**Figure 4:**
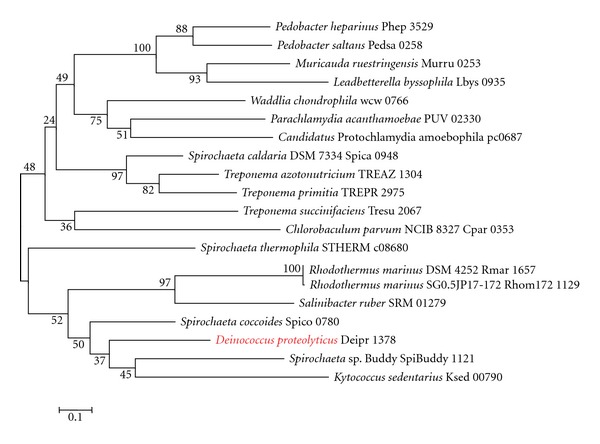
Phylogenetic relationship between *Deinococcus proteolyticus* dihydrodipicolinate reductase and related proteins. Multiple alignment was obtained using the top 20 amino acid sequences of the BLASTp search result for *D. proteolyticus* dihydrodipicolinate reductase (Deipro 1378), as based on the KEGG database. The maximum-likelihood tree was constructed using MEGA software version 5 [12]. The WAG model was used as the amino acid substitution model. The nearest neighbor interchange was used for the maximum-likelihood heuristic method. The *γ*-distributed rate was considered, and the number of discrete *γ* categories was 3. Bootstrap analysis was performed with 100 replicates. Red indicates *D. proteolyticus*.

Interestingly, *D. proteolyticus* was found to have the genes for lysine biosynthesis through the DAP pathway (Table 2). *D. proteolyticus* has 2 diaminopimelate decarboxylases, namely, Deipro 0627 and Deipro 1375 (Table 2), which are structurally different from each other. Because Deipro 1375 forms a gene cluster with other genes for lysine biosynthesis through the DAP pathway, we used Deipro 1375 as a query sequence in the BLASTp search. Each phylogenetic tree based on diaminopimelate decarboxylase (Figure 1), ll-diaminopimelate aminotransferase (Figure 2), dihydrodipicolinate synthase (Figure 3), and dihydrodipicolinate reductase (Figure 4) showed that the *D. proteolyticus* enzyme is closely related to that of the genera *Kytococcus* (a member of Actinobacteria) and *Spirochaeta* (a member of Spirochaetes) (Figures 1−4). The 3 phyla Actinobacteria, *Deinococcus-Thermus*, and Spirochaetes do not form a monophyletic lineage in the phylogenetic tree, as based on genomewide comparative studies [14]. In addition, the 4 genes encoding diaminopimelate decarboxylase, ll-diaminopimelate aminotransferase, dihydrodipicolinate synthase, and dihydrodipicolinate reductase are clustered in each genus (Figures 1−4). Thus, these findings strongly suggested that a DNA fragment including the 4 *D. proteolyticus* genes was horizontally transferred from a phylogenetically distant organism. This horizontal transfer event may have induced the loss of the genes for lysine biosynthesis through the AAA pathway in *D. proteolyticus*.

## Supplementary Material

Phylogenetic relationships among enzymes related to lysine biosynthesis through the AAA pathway.

## Figures and Tables

**Table 1:** Genes for lysine biosynthesis through the *α*-aminoadipate pathway in the *Deinococcus-Thermus* phylum.

Organism	Enzyme 1	Enzyme 2	Enzyme 3	Enzyme 4	Enzyme 5	Enzyme 6	Enzyme 7	Enzyme 8	Enzyme 9	Enzyme 10
*Thermus thermophilus* HB27	*TTC0043*	*TTC1012*	*TTC1393*	*TTC1396*	TTC1541*	TTC1542*	TTC1543*	TTC1546*	TTC1547*	TTC1550*
*Thermus thermophilus* HB8	*TTHA0411*	*TTHA1378*	*TTHA1755*	*TTHA1757*	TTHA1903*	TTHA1904*	TTHA1907*	TTHA1910*	TTHA1911*	TTHA1914*
*Thermus scotoductus*	*TSC_c05810*	*TSC_c20650*	*TSC_c03550*	*TSC_c3520*	TSC_c01940*	TSC_c01930*	TSC_c01920*	TSC_c01890*	TSC_c01880*	TSC_c01850*
*Meiothermus ruber*	*Mrub_0871*	*Mrub_2738*		*Mrub_0027*	Mrub_2721*	Mrub_2723*	Mrub_2724*	Mrub_2727*	Mrub_2728*	
*Meiothermus silvanus*	*Mesil_2567*	*Mesil_1337*	*Mesil_0348*	*Mesil_0347*	Mesil_0435*	Mesil_0436*	Mesil_0438*	Mesil_0441*	Mesil_0442*	
*Oceanithermus profundus*		*Ocepr_1387*	Ocepr_1797*	Ocepr_1798*	Ocepr_1796*	Ocepr_1788*	Ocepr_1784*	Ocepr_1781*	Ocepr_1780*	Ocepr_1779*
*Marinithermus hydrothermalis*		*Marky_1533*	Marky_0665*	Marky_0663*	Marky_0666*	Marky_0667*	Marky_0668*	Marky_0671*	Marky_0672*	Marky_0673*
*Deinococcus radiodurans*		*DR_1674*	*DR_0794*	*DR_1413*	*DR_1420*	*DR_0963*	*DR_2194*	*DR_1614*	*DR_1610*	*DR_1238*
*Deinococcus geothermalis*	*Dgeo_2084*	*Dgeo_1458*	*Dgeo_1416*	*Dgeo_1391*	*Dgeo_0678*	*Dgeo_0685*	Dgeo_1151*	Dgeo_1154*	Dgeo_1156*	*Dgeo_1257*
*Deinococcus deserti*		*Deide_09240*	*Deide_16910*	*Deide_17960*	*Deide_10430*	*Deide_10350*	Deide_13430*	Deide_13460*	Deide_13470*	*Deide_13980*
*Deinococcus maricopensis*	*Deima_0046*	*Deima_1545*	*Deima_2454*	*Deima_2593*	Deima_1346*	Deima_1349*	Deima_1350*	Deima_1353*	Deima_1355*	Deima_1358*
*Deinococcus proteolyticus*	*Deipr_0213*									
*Truepera radiovictrix*		*Trad_2841*	Trad_1401*	Trad_1404*	Trad_1399*	Trad_1395*	Trad_1392*	Trad_1390*	Trad_1389*	Trad_1388*

Enzyme 1, *α*-aminoadipate aminotransferase.

Enzyme 2, Homoisocitrate dehydrogenase.

Enzyme 3, LysW-*γ*-l-lysine aminotransferase.

Enzyme 4, LysW-*γ*-l-lysine hydrolase.

Enzyme 5, LysW-*γ*-l-*α*-aminoadipate kinase.

Enzyme 6, LysW-*γ*-l-*α*-aminoadipyl-6-phosphate reductase.

Enzyme 7, *α*-aminoadipate-LysW ligase LysX.

Enzyme 8, LysU.

Enzyme 9, LysT.

Enzyme 10, Homocitrate synthase.

*More than 3 genes are clustered.

**Table 2:** Genes for lysine biosynthesis through the diaminopimelate pathway in the *Deinococcus-Thermus* phylum.

Organism	Aspartate kinase	Aspartate-semialdehyde dehydrogenase	Dihydrodipicolinate synthase	Dihydrodipicolinate reductase	ll-diaminopimelate aminotransferase	Diaminopimelate decarboxylase
*Thermus thermophilus* HB27	*TTC0166*	*TTC0177*	*TTC0591*			
*Thermus thermophilus* HB8	*TTHA0534*	*TTHA0545*	*TTHA0957*			
*Thermus scotoductus*	*TSC_c07050*	*TSC_c08140*	*TSC_c10420*			*TSC_c10870*
*Meiothermus ruber*	*Mrub_0976*	*Mrub_1641*	*Mrub_1335*			*Mrub_0798*
*Meiothermus silvanus*	*Mesil_1711*	*Mesil_2173*	*Mesil_2308*			*Mesil_0318*
*Oceanithermus profundus*	*Ocepr_1316*	*Ocepr_1018*				*Ocepr_2076*
*Marinithermus hydrothermalis*	*Marky_1492*	*Marky_1381*	*Marky_1261*			
*Deinococcus radiodurans*	*DR_1365*	*DR_2008*				*DR_1758*
*Deinococcus geothermalis*	*Dgeo_1127*	*Dgeo_1782*				*Dgeo_0790*
*Deinococcus deserti*	*Deide_11430*	*Deide_15740*	*Deide_1p00310, Deide_3p00120, Deide_3p01100*			*Deide_12830, Deide_21880*
*Deinococcus maricopensis*	*Deima_1822*	*Deima_2680*				*Deima_2660*
*Deinococcus proteolyticus*	*Deipr_0941*	*Deipr_0985*	Deipr_1377*	Deipr_1378*	Deipr_1376*	Deipr_0627, Deipr_1375*
*Truepera radiovictrix*	*Trad_0977*	*Trad_0289*	*Trad_1893*			*Trad_0134*

*More than 3 genes are clustered.
