# Supplementary material for: Evolution of Lysine Biosynthesis in the Phylum Deinococcus-Thermus
Source: Int J Evol Biol. 2012 May 8;2012:745931. doi: 10.1155/2012/745931 (PMC3356886; doi:10.1155/2012/745931)
Supplement: Supplementary file 1 — Phylogenetic relationships among enzymes related to lysine biosynthesis through the AAA pathway. [file 745931.f1.pdf]

Supplementary Figures S1-S10.

We analyzed the distribution of each of the following 10 enzymes related to lysine biosynthesis through the AAA pathway in the *Deinococcus-Thermus* phylum: α-aminoadipate aminotransferase, homoisocitrate dehydrogenase, LysW-γ-L-lysine aminotransferase, LysW-γ-L-lysine hydrolase, LysW-γ-L-α-aminoadipate kinase, LysW-γ-L-α-aminoadipyl-6-phosphate reductase, α-aminoadipate-LysW ligase LysX, LysU, LysT, and homocitrate synthase. Homologous genes were selected on the basis of BLASTp search results by using each *T. thermophilus* enzyme for lysine biosynthesis through the AAA pathway and each *D. proteolyticus* enzyme for lysine biosynthesis through the DAP pathway. Multiple alignments were obtained using 20 amino acid sequences, with the highest to the 20th highest score by the BLASTp result. Maximum-likelihood trees were constructed using MEGA software version 5. The WAG model was used as the amino acid substitution model. The nearest neighbor interchange was used for the maximum-likelihood heuristic method. The γ-distributed rate was considered, and the number of discrete γ categories was 3. Bootstrap analysis was performed with 100 replicates. Red indicates bacteria belonging to the *Deinococcus-Thermus* phylum.

## α-aminoadipate aminotransferase and the related proteins

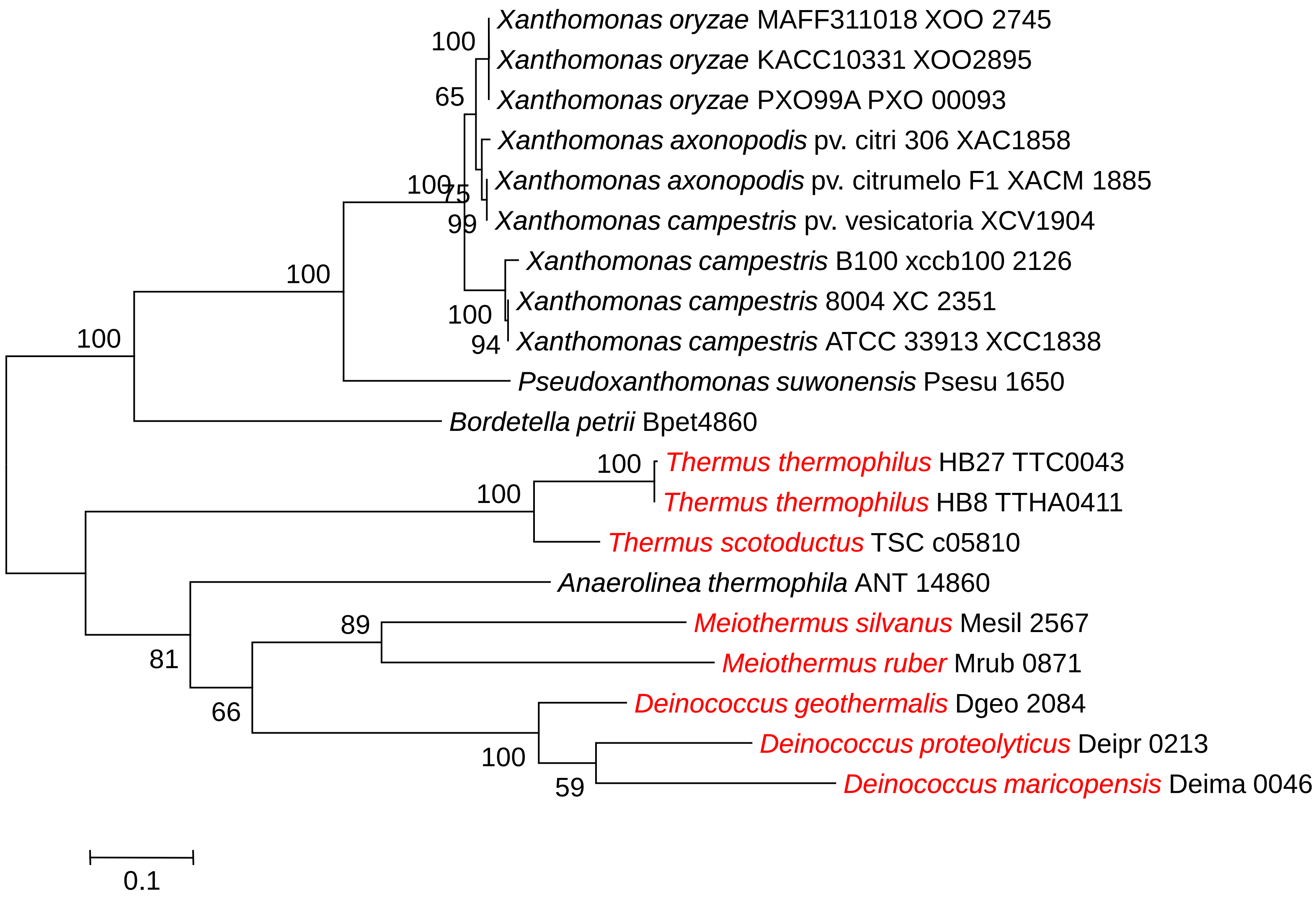


Suppl. Fig. S1

# Homoisocitrate dehydrogenase and the related proteins

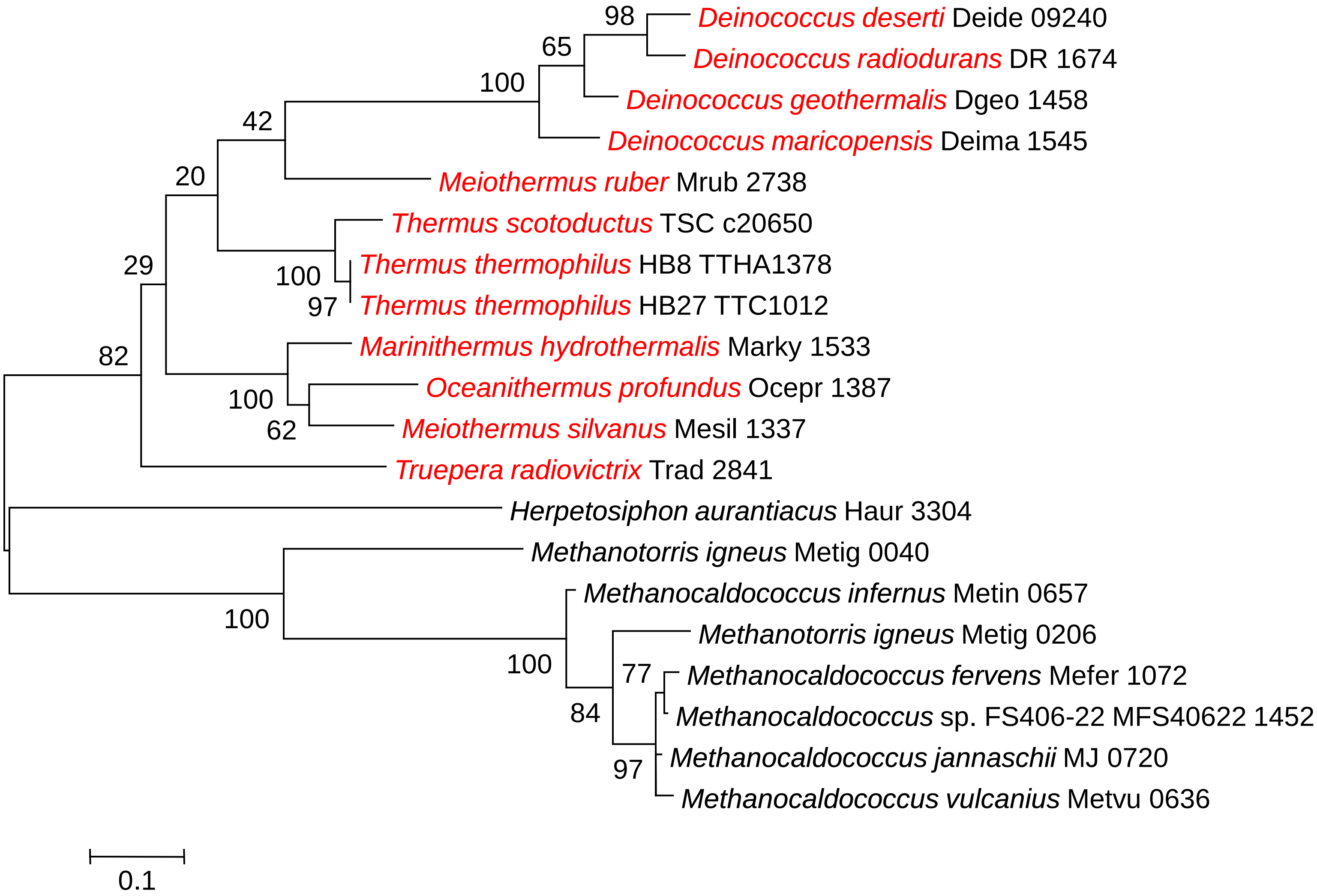


Suppl. Fig. S2

LysW-γ-L-lysine aminotransferase and the related proteins

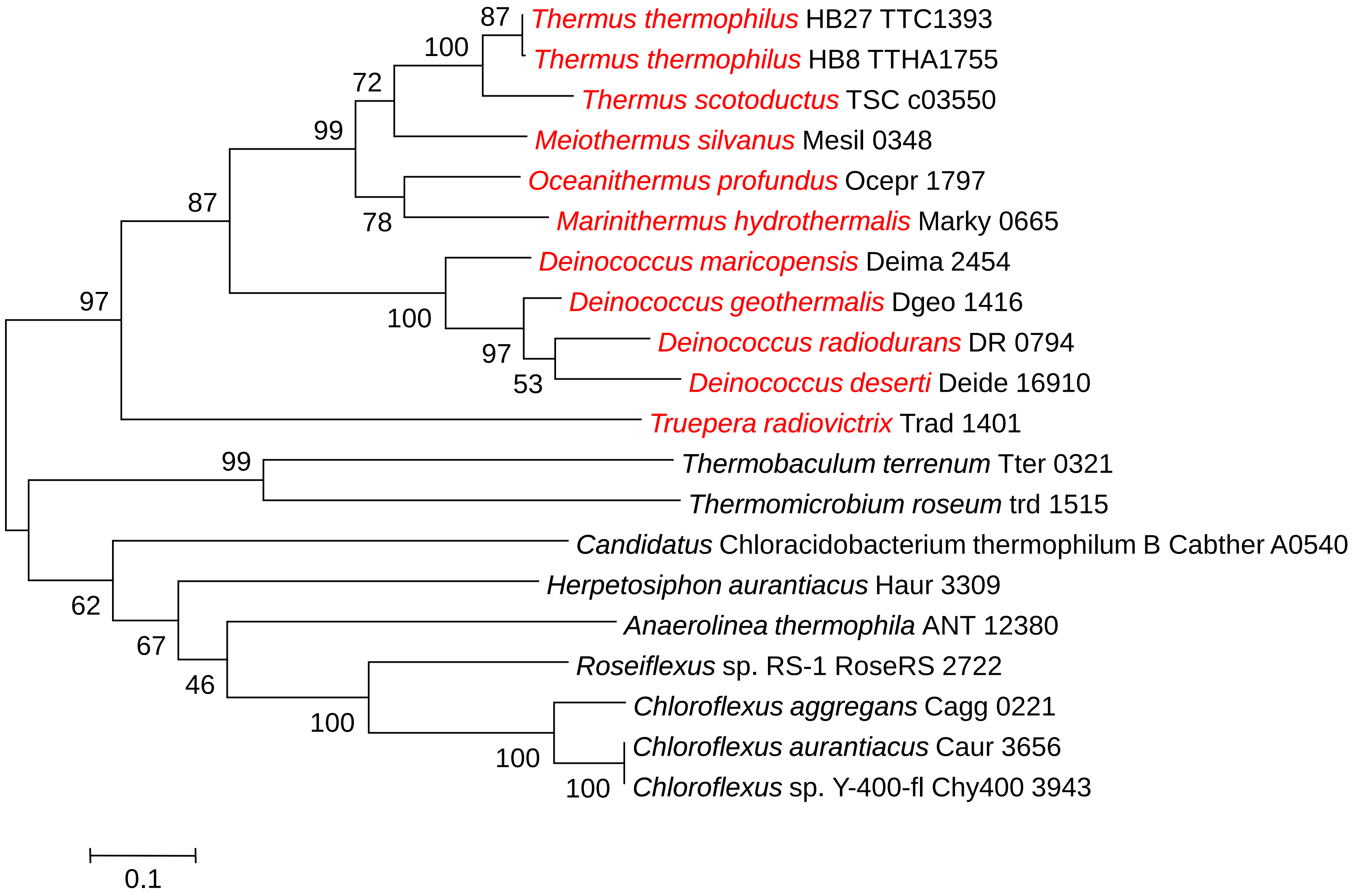


Suppl. Fig. S3

# LysW-γ-L-lysine hydrolase and the related proteins

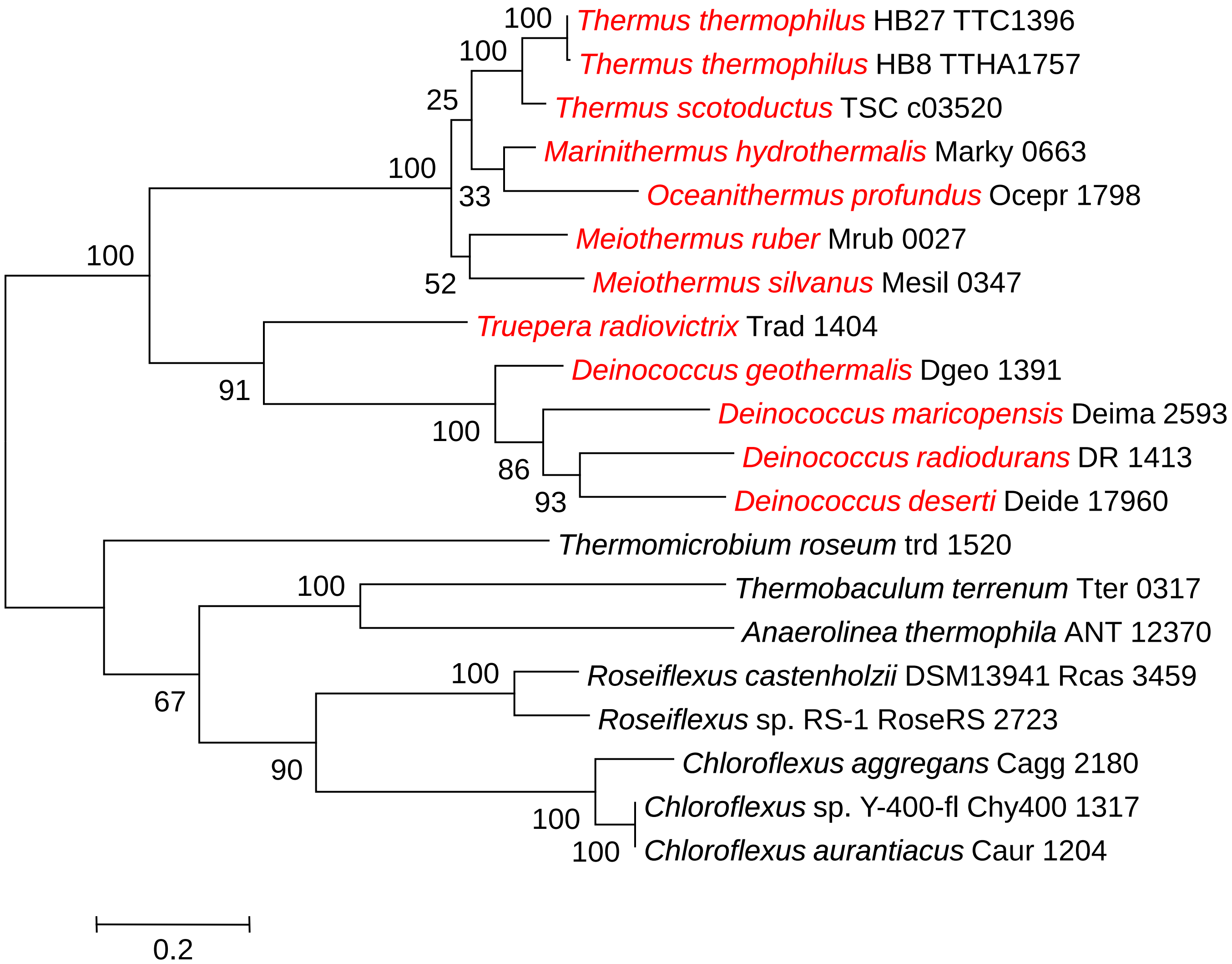


Suppl. Fig. S4

# LysW-γ-L-α-aminoadipate kinase and the related proteins

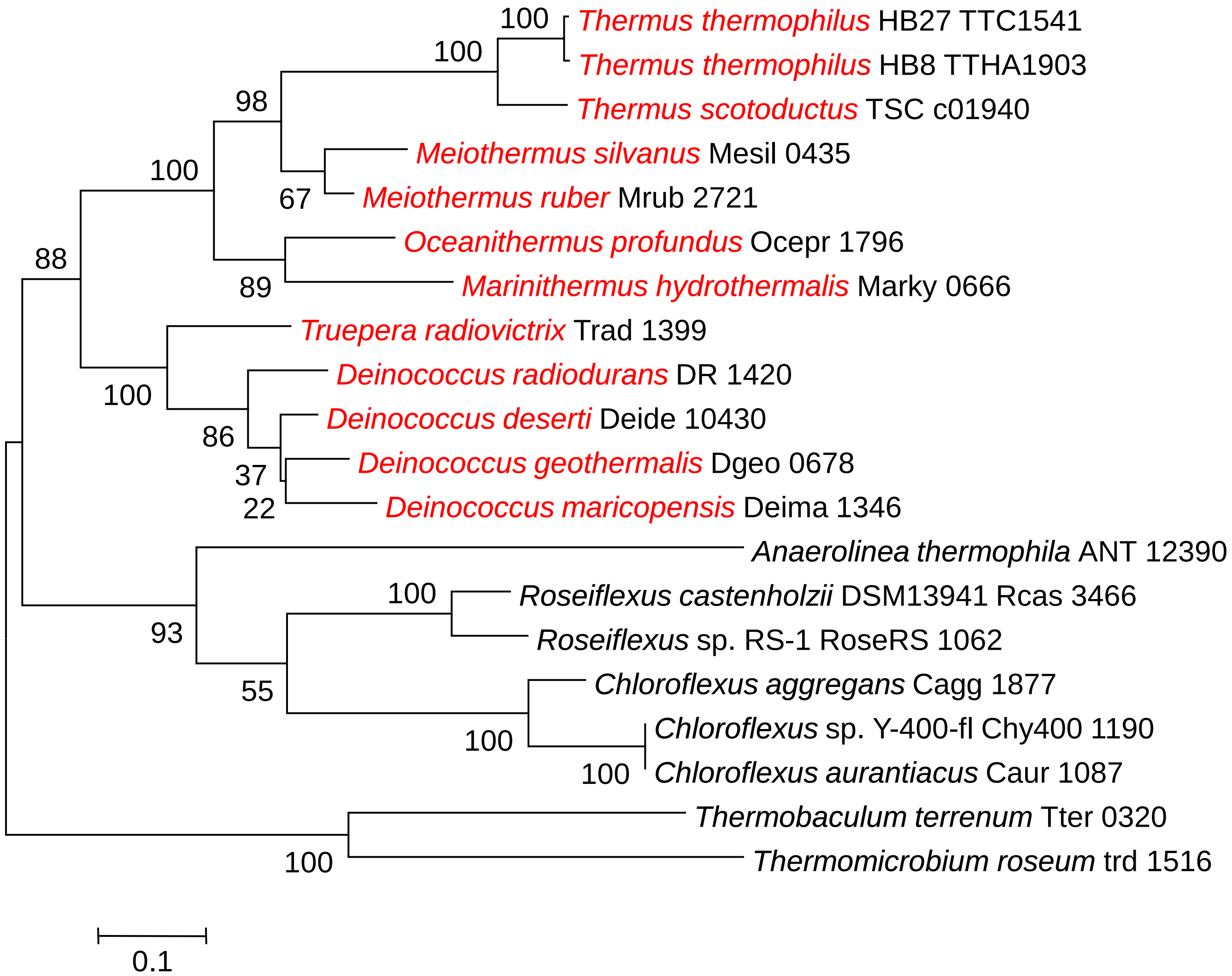


Suppl. Fig. S5

# LysW-γ-L-α-aminoadipyl-6-phosphate reductase and the related proteins

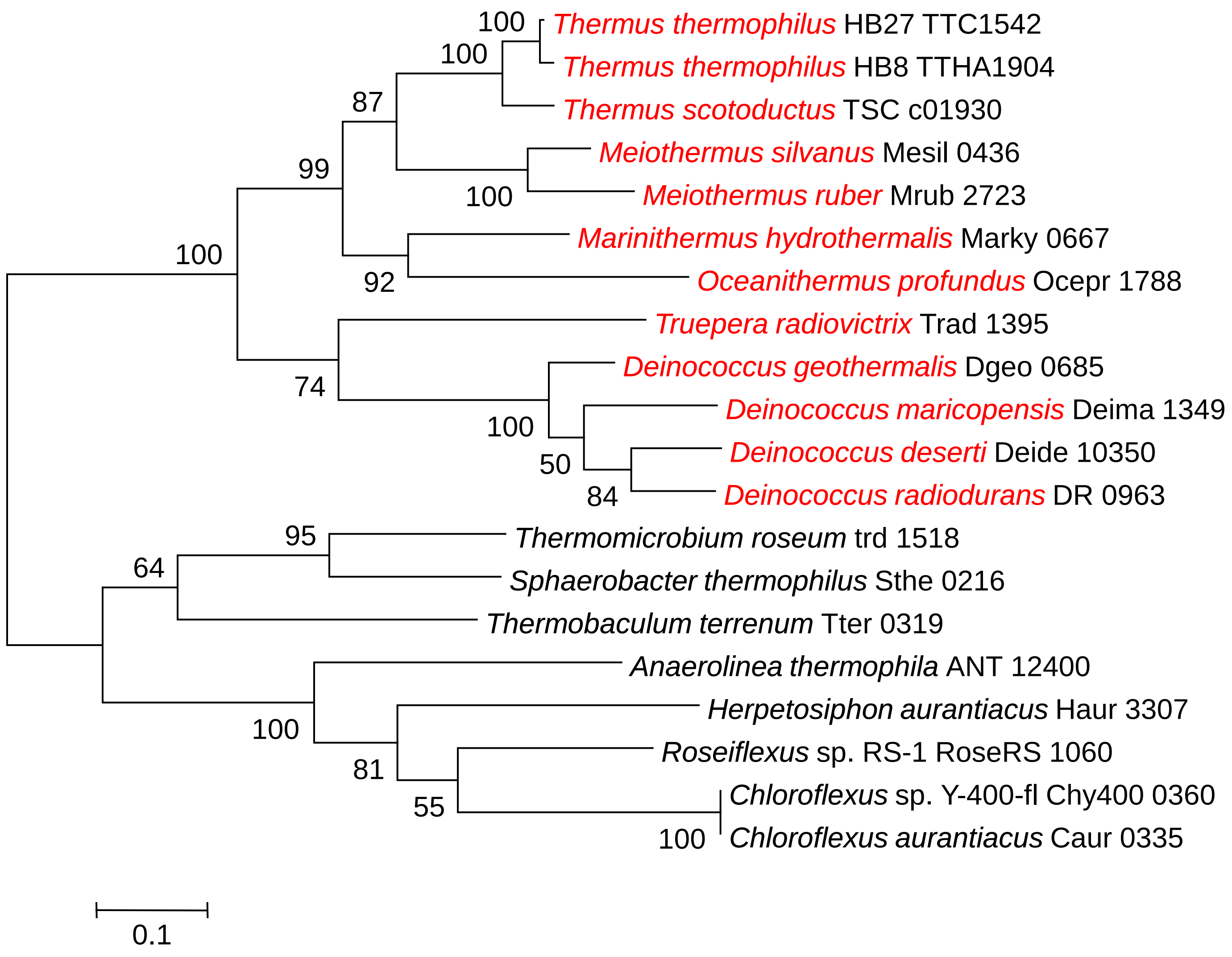


Suppl. Fig. S6

# α-aminoadipate-LysW ligase LysX and the related proteins

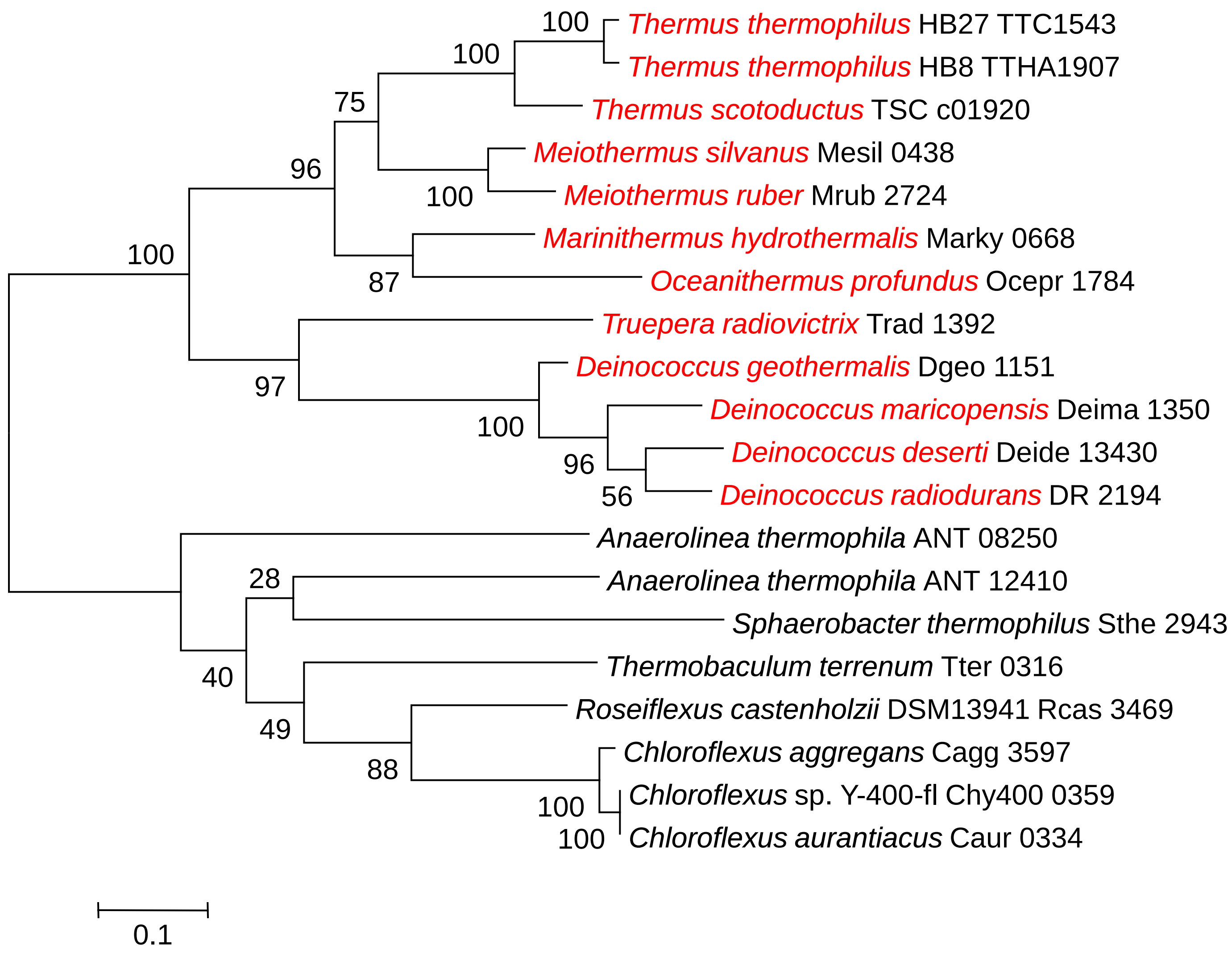


Suppl. Fig. S7

# LysU and the related proteins

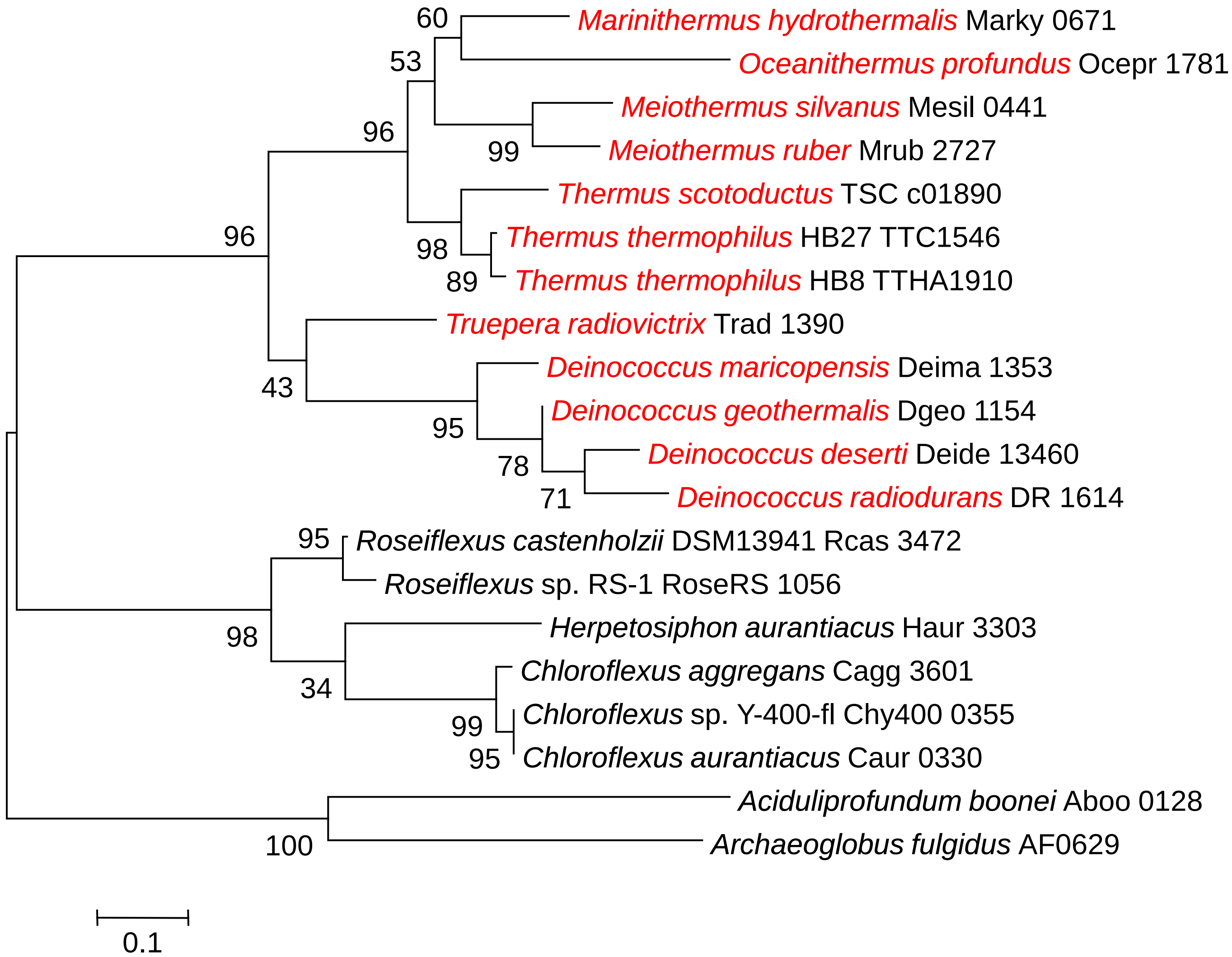


Suppl. Fig. S8

# LysT and the related proteins

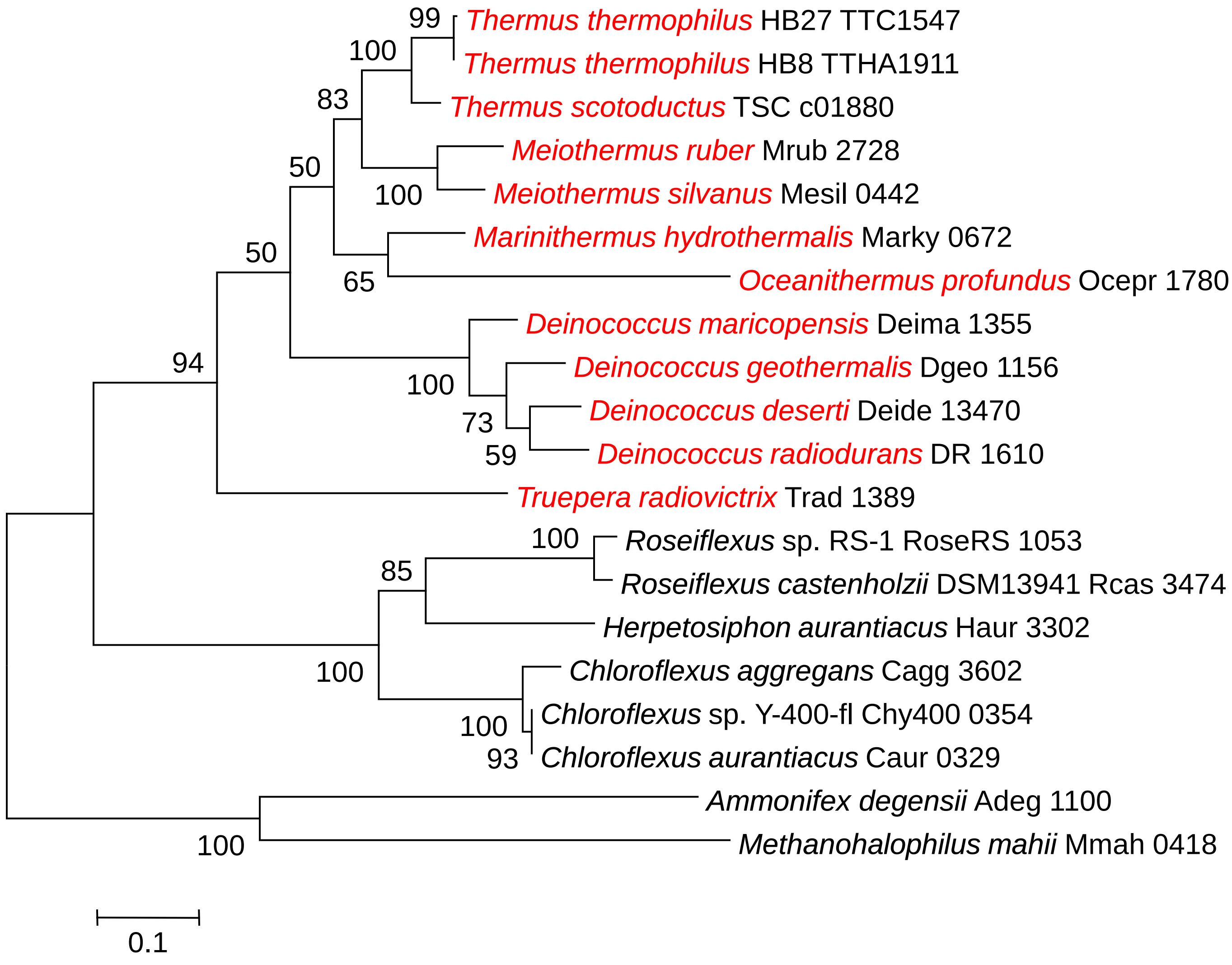


Suppl. Fig. S9

# Homocitrate synthase and the related proteins

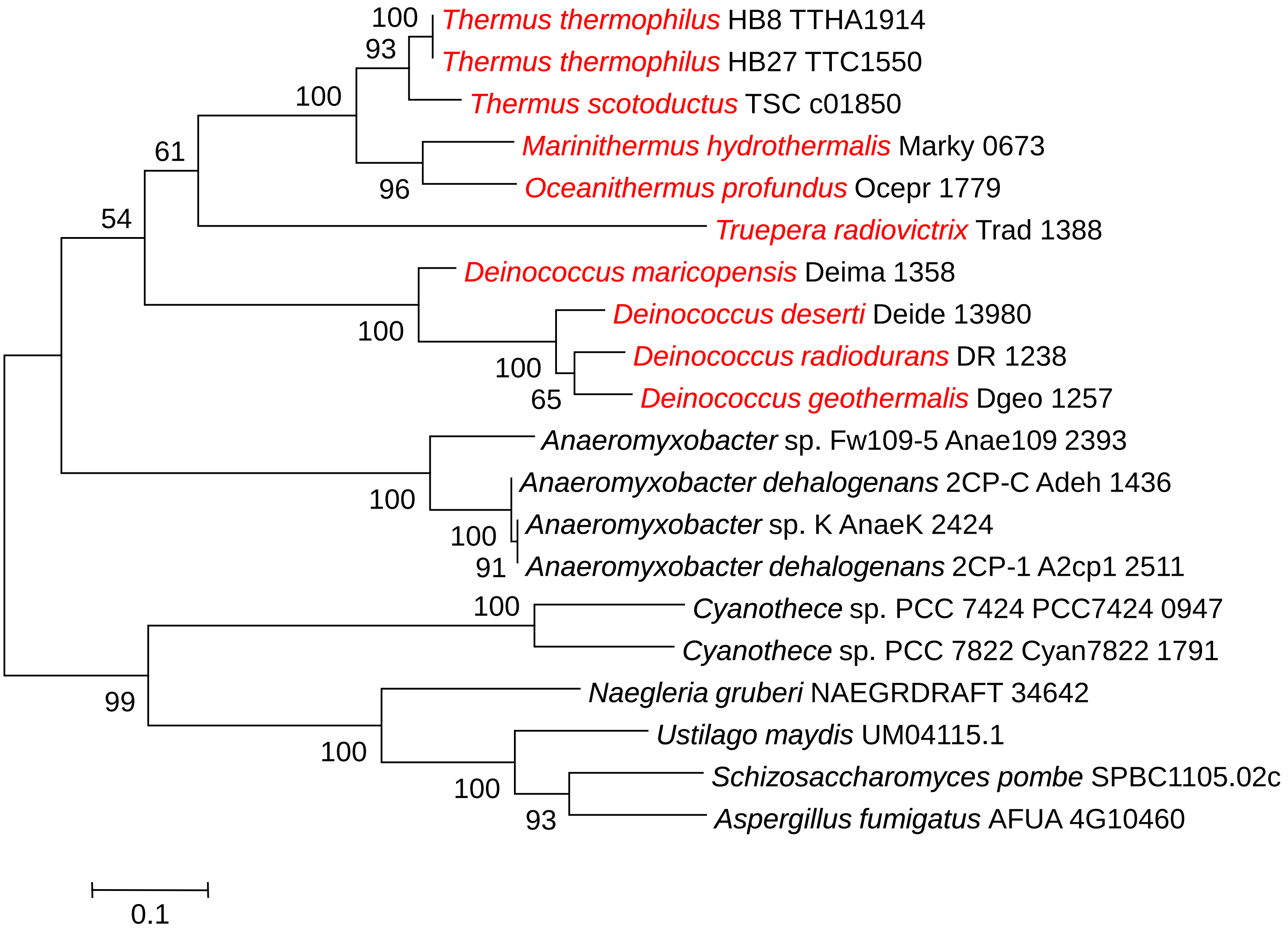


Suppl. Fig. S10
